# Action-rule-based cognitive control enables efficient execution of stimulus–response conflict tasks: a model validation of Simon task performance

**DOI:** 10.3389/fnhum.2023.1239207

**Published:** 2023-11-16

**Authors:** Yoshitaka Otani, Yoshitada Katagiri, Emiko Imai, Hisatomo Kowa

**Affiliations:** ^1^Department of Rehabilitation Science, Kobe University Graduate School of Health Sciences, Kobe, Japan; ^2^Faculty of Rehabilitation, Kobe International University, Kobe, Japan; ^3^Department of Bioengineering, School of Engineering, The University of Tokyo, Bunkyō, Japan; ^4^Department of Biophysics, Kobe University Graduate School of Health Sciences, Kobe, Japan

**Keywords:** action rule, adaptation, automatic, cognitive control, conflict, cost-saving, dorsal anterior cingulate cortex, deep-brain activity

## Abstract

**Introduction:**

The human brain can flexibly modify behavioral rules to optimize task performance (speed and accuracy) by minimizing cognitive load. To show this flexibility, we propose an action-rule-based cognitive control (ARC) model. The ARC model was based on a stochastic framework consistent with an active inference of the free energy principle, combined with schematic brain network systems regulated by the dorsal anterior cingulate cortex (dACC), to develop several hypotheses for demonstrating the validity of the ARC model.

**Methods:**

A step-motion Simon task was developed involving congruence or incongruence between important symbolic information (illustration of a foot labeled “L” or “R,” where “L” requests left and “R” requests right foot movement) and irrelevant spatial information (whether the illustration is actually of a left or right foot). We made predictions for behavioral and brain responses to testify to the theoretical predictions.

**Results:**

Task responses combined with event-related deep-brain activity (ER-DBA) measures demonstrated a key contribution of the dACC in this process and provided evidence for the main prediction that the dACC could reduce the Shannon surprise term in the free energy formula by internally reversing the irrelevant rapid anticipatory postural adaptation. We also found sequential effects with modulated dip depths of ER-DBA waveforms that support the prediction that repeated stimuli with the same congruency can promote remodeling of the internal model through the information gain term while counterbalancing the surprise term.

**Discussion:**

Overall, our results were consistent with experimental predictions, which may support the validity of the ARC model. The sequential effect accompanied by dip modulation of ER-DBA waveforms suggests that cognitive cost is saved while maintaining cognitive performance in accordance with the framework of the ARC based on 1-bit congruency-dependent selective control.

## 1 Introduction

### 1.1 General background

The selection of information important for appropriate goal-directed behaviors while ignoring irrelevant interfering or conflicting information is an essential cognitive skill. Under laboratory conditions, this capacity is often measured using Simon tasks, stimulus–response conflict (SRC) tasks where subjects are asked to produce relevant responses consistent with presented symbol information while suppressing irrelevant responses consistent with location (spatial) information when location and symbol information are incompatible (Simon, 1969; Lu and Proctor, 1995; Leuthold, 2011; Welch and Seitz, 2013). During the Simon task, irrelevant location-consistent responses are produced automatically prior to relevant symbol-consistent responses (Ivanoff, 2003; Bardi et al., 2015). Hence, the irrelevant responses must be suppressed for correct performance, resulting in response conflict (Hübner and Mishra, 2013). However, this conflict resolution mechanism enhances the cognitive load, which can lead to mental fatigue and performance errors (Marcora et al., 2009; Hornsby, 2013; Van Cutsem et al., 2017; Benoit et al., 2019; Díaz-García et al., 2021; Salomone et al., 2021).

One potential mechanism for reducing the cognitive load imposed by conflict monitoring is stimulus–response (SR) mapping for automatic goal-directed responses. It is well-known that effortful cognitive processing can be replaced under certain conditions by automatic processing (Schneider and Chein, 2003; Grossi and Coch, 2005; Verbruggen and Logan, 2009; D’Angelo et al., 2013; Spruyt and De Houwer, 2017; Anderson, 2018; Norris et al., 2018; Fabio et al., 2019), including through heuristics (Shah and Oppenheimer, 2008; Gigerenzer and Gaissmaier, 2011; Korn and Bach, 2018; Meinert and Krämer, 2022), for cost-saving while still maintaining relevant task goals. This transfer is supported by the highly flexible medial prefrontal cortex (mPFC) (Conen and Padoa-Schioppa, 2015; Rustichini and Padoa-Schioppa, 2015; Klein-Flügge et al., 2016; Rich and Wallis, 2016; Padoa-Schioppa and Conen, 2017; Ballesta and Padoa-Schioppa, 2019; Shi et al., 2022). A simple selection-rule-based cognitive processing scheme based on relevant SR mapping is an example. As the map is temporarily stored in the inferior frontal gyrus, the response to a Simon stimulus is automatically guided to the task goal without cognitive computations. This simple scheme has been formulated mathematically as the Theory of Event Coding (Hommel et al., 2001; Hommel, 2019), and numerous studies have provided evidence for the Theory of Event Coding in conflict adaptation (Clayson and Larson, 2011; Larson et al., 2011, 2012; Clawson et al., 2013; Duthoo et al., 2014; Huber-Huber and Ansorge, 2018). However, it is still debated whether conflict adaptation works in actual conflict tasks (Chen et al., 2009; Schmidt et al., 2015; Schmidt, 2019) when the tendency for irrelevant responses is strong, or whether these irrelevant responses must still be suppressed by conflict monitoring.

### 1.2 Proposal of action-rule-based cognitive control (ARC)

Here, we propose a novel action-rule-based cognitive control (ARC) model as an alternative to the conventional conflict adaptation framework for executing SRC tasks. In this model, the dorsal anterior cingulate cortex (dACC) acts as a primary conductor by integrating the outputs of intrinsic large-scale networks such as the salience, central executive, and default-mode networks (Bressler and Menon, 2010; Schulz et al., 2011; Sheth et al., 2012; Lanius et al., 2015; Heilbronner and Hayden, 2016; Wang et al., 2016; Aben et al., 2020). This study aimed to investigate whether conflict resolution can be accomplished in accordance with the model. To this end, we started by explaining the ARC model in detail, followed by hypotheses and predictions based on a conventional neural network model to understand how the ARC model is reflected in the actual brain, finally using a simple computational model for making quantitative predictions to evaluate experimental data.

#### 1.2.1 Concept of the ARC model

**Figure 1:**
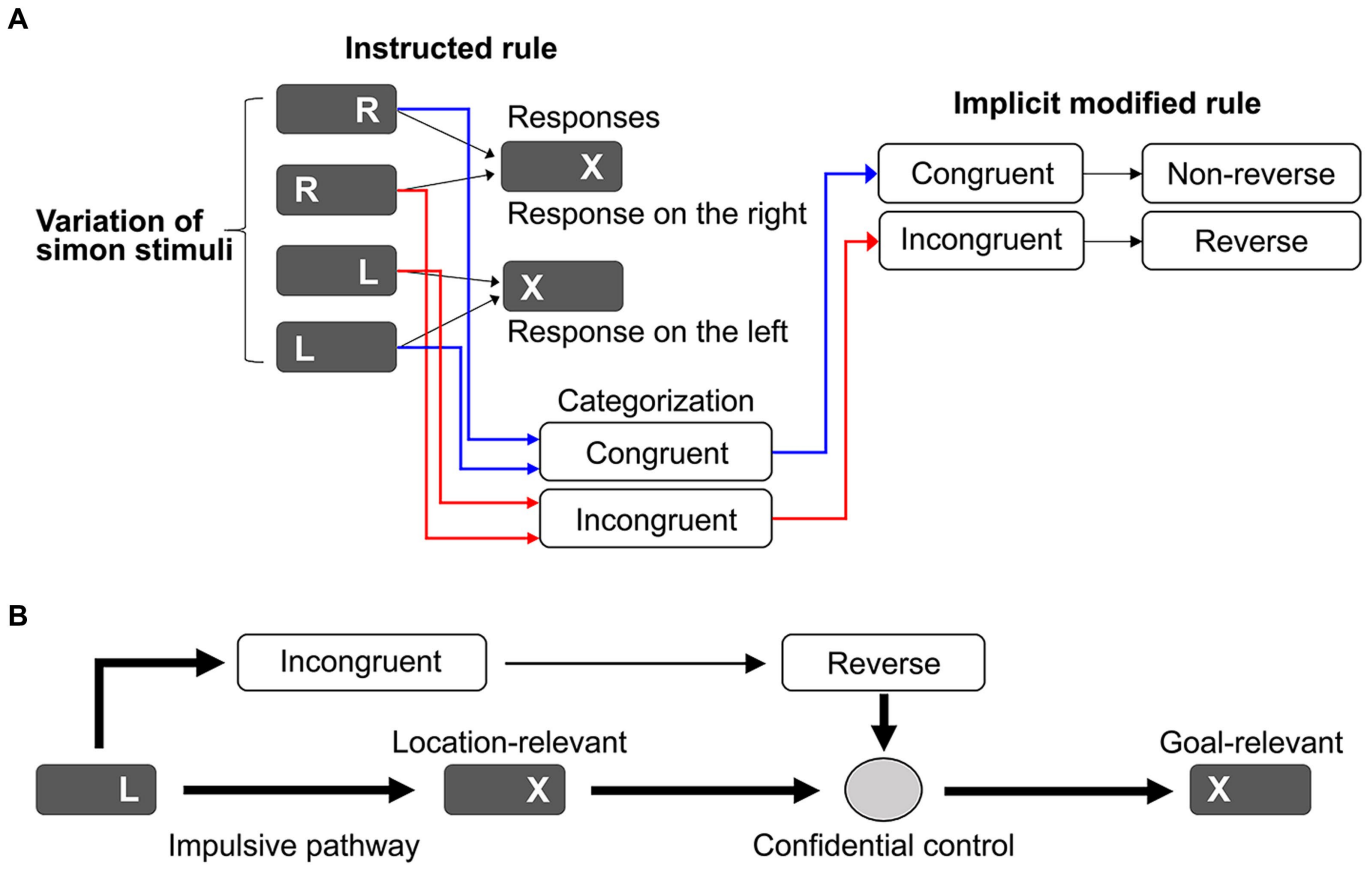
**(A)** Schematic diagrams for explaining the action-rule-based cognitive control model. Conventionally, four types of Simon stimulus variations have been linked to the task-relevant responses (responses on the right/left). In our model, these four types are simply categorized as congruent or incongruent. These states are linked to the action rule where behavioral adaptation is switched from nonreverse for the congruent condition to reverse for the incongruent condition. **(B)** A specific application for the Simon task to guide impulsive responses to the task goal, where behavioral adaptation is switched from nonreverse for the congruent condition to reverse for the incongruent condition to guide impulsive responses to the task goal. The behavioral adaptation is conducted based on confidence in the action rule.

In general, the Simon task is distinguished from other conflict tasks (Kleinsorge, 2021) by dimensional overlap and taxonomy (Kornblum, 1994; Scerrati et al., 2017). The Simon task generates a dimensional overlap between two response sets and the task-irrelevant stimulus location dimension. This dimensional overlap causes a SRC. The Theory of Event Coding based on stimulus–response mapping is expected to efficiently resolve the SRC (Hommel et al., 2001; Hommel, 2019); however, the effect of SR mapping may be disrupted by fast, task-irrelevant responses to stimulus location. Here, we propose an ARC model that utilizes location-relevant responses (Figure 1A). Simon stimuli include symbols and locations that define four stimulus types. We assumed that Simon stimuli can be simply categorized as congruent or incongruent (Ashby and Spiering, 2004; Poldrack and Rodriguez, 2004; Poldrack and Foerde, 2008). Location-relevant responses are promptly guided to the task goal if adequately adapted based on the simple rule that responses are horizontally reversed for the incongruent condition but non-reversed for the congruent condition. This simple rule yields a novel task set accompanied by a category–action rule map. Figure 1B shows a practical implementation of the ARC model. The task-irrelevant response generated by the incongruent Simon stimulus is promptly corrected based on the category–action map and then executed. Such cognitive control relies on the confidence that the category–action rule will guide automatic responses to the task goal, thereby avoiding SRC and reducing cognitive load (cost saving).

Moreover, the informational entropy is reduced from 2 bits (location [right/left] × symbol [R/L]) to 1 bit (congruent/incongruent). The generation of automatic location-relevant responses assists in this information entropy reduction. Specifically, an impulsive response to the location information of the presented stimulus causes a reduce in the information-entropy in SR mapping. According to information theory (Shannon, 1948), the processing cost reduction, as evaluated by information entropy reduction, is


(1) $$H=-\underset{i}{\sum }p_{i}\log p_{i}\text{,}$$

where pi is the occurrence probability.

Given that the degrees of freedom decrease from 4 to 2 by categorization, the reduction in processing cost for determining the goal-relevant state, as evaluated by the entropy reduction, is


(2) ΔH=−log2,

where the occurrence probability of the final state is assumed to be unity.

The ARC model is completely conceptual and practically relies on the confidence based on metacognitive knowledge gained via experience, as there are no explicit goal representations. Therefore, we need to verify whether the model is consistent with neurophysiological observations.

Extending this cost-saving effect, we propose that the action rule can avoid the expansion of category × response combinations in conventional SR maps (Iyengar and Lepper, 2000; Reutskaja et al., 2018), thereby reducing the cognitive processing costs associated with mapped memories. Further, our model promotes dynamic regulation between impulsive and strategic cognitive control for achieving the best effort during tasks. This could allow highly accurate ballistic cognitive control beyond the speed versus accuracy trade-off (Herz et al., 2017; Vandierendonck, 2021; Mittelstädt et al., 2022).

#### 1.2.2 Computational framework for simulating the ARC model

We here provide a simple computational framework for developing the ARC model, which is necessary for experimentally demonstrating the validity of the ARC model with an appropriate Simon task. In the Simon task, the stimuli provide not only visual ambiguity from congruency between spatial and symbolic information but also temporal ambiguity from the sequence structure consisting of randomly allocated incongruent and congruent trials. We here present a framework based on the stochastic modeling of the brain, which refers to the free energy principle (FEP) (Friston, 2010).

This framework utilizes two vectors for expressing the right and left responses as

$\phi \left(\zeta =0\right)=\left(\begin{array}{c} 1\\ 0 \end{array}\right)$ (right) and $\phi \left(\zeta =1\right)=\left(\begin{array}{c} 0\\ 1 \end{array}\right)$ (left),(3)

where ζ is the parameter representing right (0) and left (1). A matrix operator is introduced as


(4) $$\Psi =\Psi^{-1}=\left(\begin{array}{cc} 0 & 1\\ 1 & 0 \end{array}\right)\text{.}$$

This operator simply provides the following reversible relationship


(5) $$\phi \left(0\right)=\psi \phi \left(1\right),\phi \left(1\right)=\psi \phi \left(0\right)\text{.}$$

**Figure 2:**
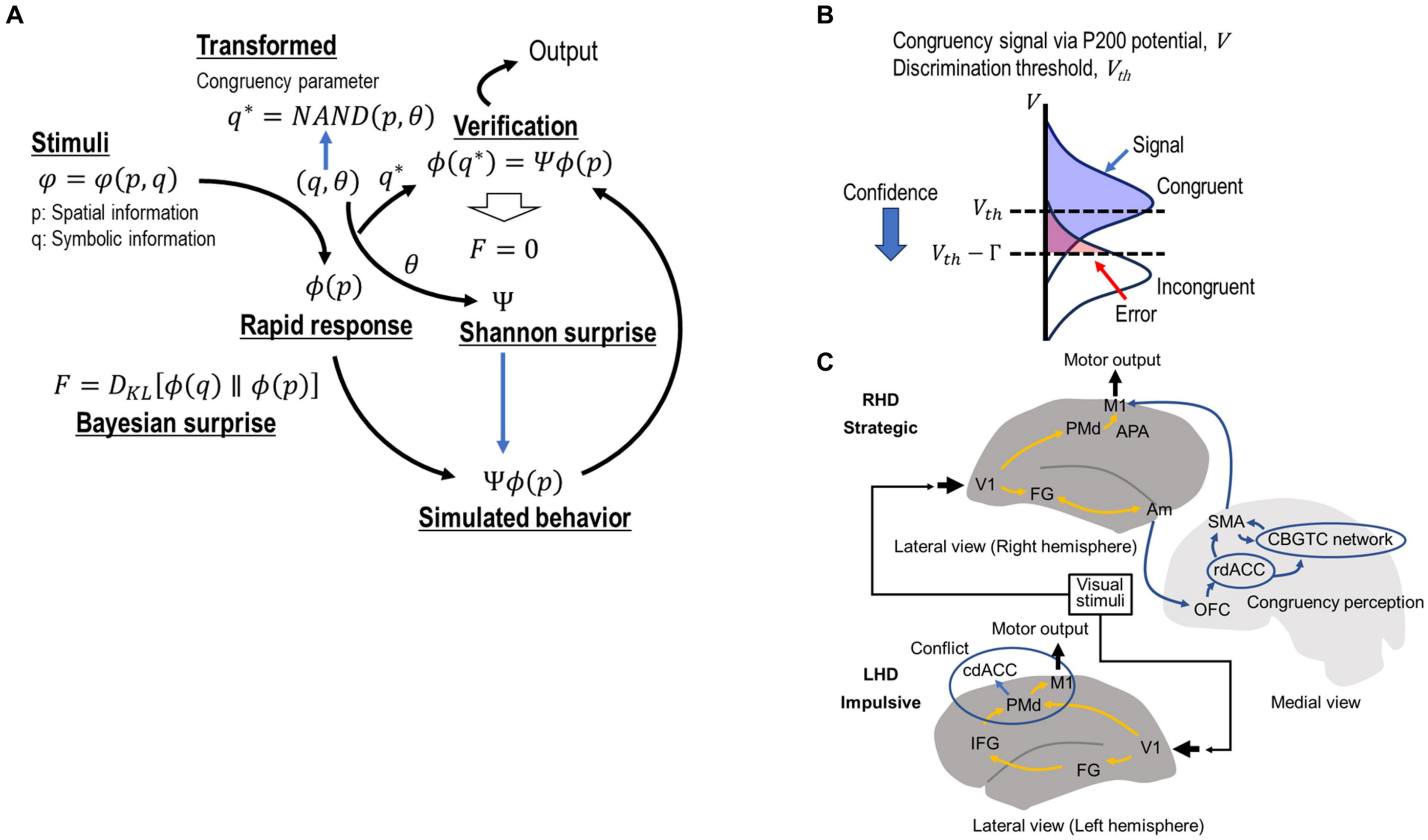
**(A)** Computational framework based on the free energy principle. Simon stimuli $\varphi =\varphi \left(p,q\right)$ (*p*/*q*, spatial/symbolic information) generate space-relevant rapid responses $\phi \left(p\right)$ . The symbolic information *q*, which can be presumed using a congruency parameter θ and spatial information *p* as $q^{*}=\mathrm{NAND}\left[\theta ,p\right]$ activates inference $Q\left(\varphi \right)$ based on the internal model before semantic processing. The inference error $D_{KL}\left[\phi \left(q^{*}\right)∥P\left(\phi \left(p\right)\right)\right]$ can be dramatically reduced as $D_{KL}\left[\phi \left(q^{*}\right)∥P\left(\Psi \phi \left(p\right)\right)\right]$ = $D_{KL}\left[\phi \left(q^{*}\right)∥\phi \left(q\right)\right]$ using virtual behavioral adaptation $\Psi \phi \left(p\right)$ as active inference. The modified response code via active inference is executed by confirming the consistency of active inference with the goal as $\phi \left(q\right)=\Psi \phi \left(p\right)=\phi \left(q^{*}\right)$ . **(B)** Sensitivity modulation for enhancing active inference. Active inference is promoted based on congruency parameter θ , which is assumed to be discriminated on the basis of a threshold $V_{th}$ for the congruency-related visual signal *V*. The inference is enhanced by mediating the threshold as $V_{th}^{*}=V_{th}-{\left(-1\right)}^{\theta }\Gamma$ , where Γ is a constant. This equation also implies that repeating congruent trials reinforces the normal state. **(C)** The functional architecture of the cognitive control system based on visual information. The scheme presents the known functions of dissociable dorsal and ventral visual information processing pathways, lateralized functions of the fusiform gyrus (FG), and contributions of the anterior cingulate cortex (ACC) in impulsive and strategic decision-making. The left FG (part of the ventral pathway) analyzes symbolic information. In the conventional dual root model, this symbolic information is transferred to the DLPFC, which potentially interferes with the dorsal visual information pathway at the response level, causing response conflict. However, in the ARC model, symbolic information is transferred to the rostral part of the dACC via the orbitofrontal cortex. The rostral dACC can modify the initial response code from the dorsal pathway via the CBGTC network with the involvement of the SMA. This modification corresponds to active inference. The amygdala evaluates the cognitive load for each congruency and introduces bias for distinguishing between congruence and incongruence. The dACC reaches the final decision for providing goal-relevant responses by confirming that the adapted response is consistent with the symbolic information (q). Note that semantic information is not necessarily required in this comparison stage. IFG, inferior frontal gyrus; DLPFC, dorsolateral prefrontal cortex; PMd, dorsal premotor cortex; dACC, dorsal anterior cingulate cortex; rdACC, rostral dorsal anterior cingulate cortex; cdACC, caudal dorsal anterior cingulate cortex; SMA, supplementary motor area; OFC, orbitofrontal cortex; M1, primary motor cortex; V1, primary visual area; CBGTC, cortico-basal ganglia-thalamo-cortical.

We further introduced a scalar parameter θ for expressing congruency as θ = 0 for congruence and θ = 1 for incongruence. Using the above formula, Figure 2A represents the cognitive processes occurring during the Simon task based on the FEP. The Simon stimuli containing the spatial information *p* and symbolic information *q* as $\varphi =\varphi \left(p,q\right)$ immediately generates rapid responses compatible with the spatial information generated as $\phi \left(p\right)$ . To determine the conditional probability of the stimulus state φ occurring under the condition $\phi \left(p\right)$ , the brain generates an inference as $Q\left(\;\varphi \right)$ , accompanied by an inference error using the Kullback–Leibler divergence formalism as $D_{KL}\left[Q\left(\varphi \right)∥P\left(\varphi |\phi \left(\varphi \right)\right)\right]$ . In the FEP framework, free energy is defined as


(6) $$F=D_{KL}\left[Q\left(\varphi \right)∥P\left(\varphi ,\phi \left(\varphi \right)\right)\right]\text{.}$$

Considering the conditioning probability formula $P\left(A,B\right)=P\left(A|B\right)P\left(B\right)$ , the free energy is expressed as


(7) $$F=D_{KL}\left[Q\left(\varphi \right)∥P\left(\varphi |\phi \left(\varphi \right)\right)\right]-\mathrm{logP}\left(\phi \left(\varphi \right)\right)\text{,}$$

where the first term is the Bayesian surprise, followed by the Shannon surprise. The brain aims to minimize the free energy, which leads to inference with the highest likelihood. For the congruent condition p=q the free energy is zero. However, for the incongruent condition p≠q , the free energy increases due to the large inference error corresponding to the Bayesian surprise and the rare discomfort corresponding to the Shannon surprise as


(8) $$F=D_{KL}\left[Q\left(\phi \left(q\right)\right)∥P\left(\varphi |\phi \left(p\right)\right)\right]-\mathrm{logP}\left(\phi \left(p\right)\right)\text{.}$$

However, this free energy is readily decreased by the active inference framework (Friston et al., 2017), where the brain virtually mediates a rapid response $\Psi \phi \left(p\right)$ , supported by the congruency signal θ. The free energy becomes


$$\begin{array}{c} F=D_{KL}\left[\phi \left(q\right)∥\Psi \phi \left(p\right)\right]-\mathrm{logP}\left(\Psi \phi \left(p\right)\right)\\ =D_{KL}\left[\phi \left(q\right)∥\phi \left(q\right)\right]-\mathrm{logP}\left(\phi \left(q\right)\right) \end{array}$$

where, the lower limit of the free energy is Shannon surprise, and indicating that the best possible case where errors are minimized for the incongruence.

The brain confirms that the adapted behavior $\Psi \phi \left(p\right)$ is equal to $\phi \left(q\right)$ for p≠q (incongruence), thereby providing goal-relevant responses without response conflicts. Here, the Bayesian surprise term can be reduced by modifying the internal model so that the semantic definition can be exchanged between the right and left as $q\to \bar{q}$ , where means R→L,L→R . Such Bayesian inference term reduction minimizes free energy. However, the brain metacognitively recognizes that the space-compatible response $\phi \left(p\right)$ is irrelevant for the final task goal $\phi \left(q\right)$ thus, such Bayesian inference is invalid for the Simon task and thereby minimized. Consequently, active inference is enhanced.

In cognitive processing following the ARC model, active inference is enhanced by adjustment the sensitivity to the incongruence.

Here, we assume that the congruency is indirectly estimated from the sensory signal V based on an appropriate discrimination threshold


$$V_{th}$$

as


(9) $$V>V_{th}\Rightarrow \theta =1\;\left(\mathrm{congruence}\right);V<V_{th}\Rightarrow \theta =0\left(\mathrm{incongruence}\right)\text{,}$$

where the sensory signal


$$M\left(V,\theta \right)$$

is expressed considering the Gaussian distribution

$$\sigma_{\theta }$$

as


(10) $$M\left(V,\theta \right)=\frac{V_{\theta }}{\sigma \sqrt{2\pi }}\exp\left[-\frac{1}{2}{\left(\frac{V-V_{\theta }}{\sigma_{\theta }}\right)}^{2}\right]\text{,}$$

where $V_{0}$ and $V_{1}$ ( $V_{1}>V_{0}$ ) are the peak values of the sensory signals for congruence and incongruence, respectively. Hence, the sensitivity to incongruence and discrimination reliability is evaluated as


(11) $$S\left(\theta =1\right)=1/\left(1+\frac{\kappa -1}{\kappa }\overset{V_{th}}{\underset{-\infty }{\int }}M\left(V,0\right)dV/\overset{\infty }{\underset{V_{th}}{\int }}M\left(V,1\right)dV\right)\text{,}$$

where κ is the frequency ratio between incongruence and congruence.

This equation suggests that decreasing the discrimination threshold improves sensitivity to congruence. Such top-down mediation of the sensory cortex is valid for the congruent condition because there is no risk of accepting the rapid response $\phi \left(p\right)$ as the final approved behavior for the task. Hence, the brain will improve the sensitivity to incongruence as


(12) $$S\left(\theta =0\right)=1/\left(1+\frac{\kappa }{1-\kappa }\overset{\infty }{\underset{V_{th}}{\int }}M\left(V,1\right)dV/\overset{V_{th}}{\underset{-\infty }{\int }}M\left(V,0\right)dV\right)\text{,}$$

Biases due to sequence effects can be expressed as adjustments to the active-discrimination threshold. In such an adjustment, sensitivity increases when congruent sequences are provided. Conversely, sensitivity is reduced for incongruent sequences. If the consistency does not change after the sensitivity was adjusted, error responses do not occur immediately. However, because of the potentially increased false detection probability, error responses are likely to occur in the sequence in which consistency switches. Under a condition of repeated trials with the same congruency, such threshold mediation is accumulated by increasing the number of repeated trials as


(13) $$V_{th}=\rho \left(\kappa \right)\left\{V_{th}+{\left(-1\right)}^{\theta }\Gamma \right\}\text{,}$$

where Γ represents the threshold variation. This threshold mediation promotes reinforcement learning for maximizing sensitivity to each congruency (Colas et al., 2022). Figure 2B schematically shows this threshold mediation.

The cognitive cost is higher for incongruent trials than for congruent trials, which can be attributed to active inference. Simon stimuli provide a specific sequence where congruent and incongruent blocks are alternatively provided. Hence, the cognitive cost is high in incongruent blocks but low in congruent blocks. Such congruency-dependent cognitive cost modulation is beneficial for total cost savings: cognitive cost could be saved during the congruent trials, whereas additional cost is required only in the incongruent trials. Note the risk of such cognitive cost modulation as well as the benefit. In the incongruent trial subsequent to the congruent, active inference could be missing or incorrectly adapted by the repetition of the congruent trials. Such a situation is attributed to lower cognitive load. In contrast, in the congruent trial subsequent to the incongruent, the active inference could be erroneously applied to the congruent trial. Such opposite situation can attribute to excessive cognitive cost for active inference. Consequently, the ARC model predicts response conflicts limited to such irrelevant responses in congruent trials preceded by incongruent trial. On the other hand, predicting no conflicts for relevant responses under such as congruence sequence conditions.

#### 1.2.3 Integration of a computational framework with an actual brain model for experimental predictions

To examine how the ARC model works in the actual brain, we integrated the computational framework with a conventional brain model (Figure 2C).

In general, visual information is processed via dissociable dorsal and ventral pathways. The dorsal pathway primarily processes location information, while the ventral pathway focuses on symbolic information (Sakai et al., 2000; Soutschek et al., 2013). Thus, location-relevant early-stage anticipatory postural adaptations (APAs) are caused, at least in part, by input from the dorsal pathway. Information processing in the dorsal pathway is faster than in the ventral pathway, so early-stage APAs are likely completely location-relevant without interference from the symbolic information processed by the ventral pathway. Hence, APA corresponds to a rapid response in the computational framework. Taken together, we first predicted that the location-compatible APA could appear at almost the same time, independently of congruency.

Next, we examined how the brain promptly detects congruency *θ* much before sematic processing to promote active inference utilizing the rapid APA response. The whole symbolic feature of Simon stimuli may be processed as a “visual form-associated” by the left fusiform gyrus (FG), which is part of the ventral pathway (Ma and Han, 2012; Carlos et al., 2019; Hildesheim et al., 2020). Such visual form-associated processing is reflected by the P200 event-related potential (ERP) component (He et al., 2019; Cao et al., 2020; Teixeira-Santos et al., 2020; Zhang et al., 2020; Hsu et al., 2021; Larionova and Martynova, 2022). In such visual form-associate FG, specific visual stimuli, such the incongruent image in the Simon task, could be weighted by the amygdala–FG circuit (Frank et al., 2019), potentially affecting the P200 amplitude. Hence, we made a second prediction: the P200 could reflect congruency.

Finally, we theoretically examined how active inference could be promoted by the dACC, which strongly supports the ARC model. The congruency information detected in the FG may be transferred to the rostral dACC via the orbitofrontal cortex (OFC), where it is promptly used for activating processes associated with active inference utilizing the early-stage APA via the supplementary motor area (SMA) (Lerner et al., 2009; Asemi et al., 2015; Diwadkar et al., 2017). We speculate that active inference accompanied by reciprocal APA changes is promoted by strategic cognitive control signals from the rostral part of the dACC under the “incongruence” signal.

The dACC may improve connectivity with the mPFC, a node of the default-mode network needed to activate the action rule, i.e., the matrix operator Ψ, as described in the computational framework and modulate the APA via the OFC–striatum pathway.

The rostral dACC may promote this processing while suppressing any other task-unrelated activities, manifesting deactivation. Hence, we made the third prediction that task-relevant responses could be accompanied by dACC deactivation, while irrelevant responses could not.

### 1.3 Aim of the current study

The current study aimed to show the validity of the ARC model through testable tasks. To this end, we devised a step-motion Simon task on the response elements essential for achieving task goals, including robust APA based on location information and congruency-based action-rule adaptation for automatically generated APA. In this study, four trial conditions were defined according to sequential congruency [Current trial (previous trial)]: C(C), C(I), I(I), and I(C), where C and I refer to congruent trial and incongruent trial.

Based on our theoretical considerations, we developed three theoretical and two experimental predictions:

Theoretical predictions that:

(1) The cognitive cost could be improved in the incongruent blocks and reduced in the congruent blocks. (2) Error risk increases with congruence change [C(I), I(C)]. Error at I(C): reactive error under low cognitive load. Error at C(I): SRC under higher cognitive load. However, due to the higher cognitive load, the error frequency could be rather low.

Experimental predictions that:

(3) APA occurs almost at the same time, independently of congruency. In other words, the APAs appear in every sequence in accordance with the spatial characteristics of the stimuli, and the latencies are comparable. (4) The ERP P200 component reflects congruency. We predicted that incongruences such as I(I) or I(C) would show a larger P200 than C(C) or C(I). (5) Appropriate task execution needs adequate dACC activity accompanied by deactivation (ER-DBA dips). We expected the ER-DBA dip to be larger in the incongruent than in the congruent condition. It was also expected that the ER-DBA dip would be more pronounced in the C(I) and I(C) sequences, in which the stimulus characteristics switch, than in the C(C) sequence, in which the same stimulus is provided.

Then, we investigated behavioral and brain responses during tasks to confirm the validity of our hypotheses and predictions. We adopted an event-related deep-brain activity (ER-DBA) method to evaluate the dynamics of dACC activity with high temporal accuracy (~300 ms) using electroencephalogram (EEG) measures of occipital alpha-2 power fluctuations (Omata et al., 2013). The ER-DBA method evaluates has been shown to reflect the activities of regions such as the dACC and ventral tegmental area (VTA) (Omata et al., 2013). This technique is characterized by its ability to assess the time course of cognitive workload via dACC activity. Behavioral and additional neurophysiological data, including acceleration (ACC), electromyogram (EMG) and event-related potentials (ERPs), were acquired to further support our model.

## 2 Methods

### 2.1 Subjects

Healthy young university and graduate students (23.8 ± 7.0 years) were recruited from Kobe International University, and 17 (4 females, 13 males) ultimately participated in the study. A The sex ratio was imbalanced, however, we considered that this would not affect experimental results, considering argument by recent studies providing opposing claims on gender difference in the Simon effect (Evans and Hampson, 2015; Stoet, 2017). All had normal vision, and none were using psychotropic agents. Prior to the study, participants were briefed on study goals and methods, and informed that they were free to withdraw at any time without penalty in accordance with the Declaration of Helsinki. All provided written informed consent for participation, and the study protocol was approved by the Ethics Committee of Kobe University Graduate School of Health Sciences (No. 885).

### 2.2 Procedure

**Figure 3:**
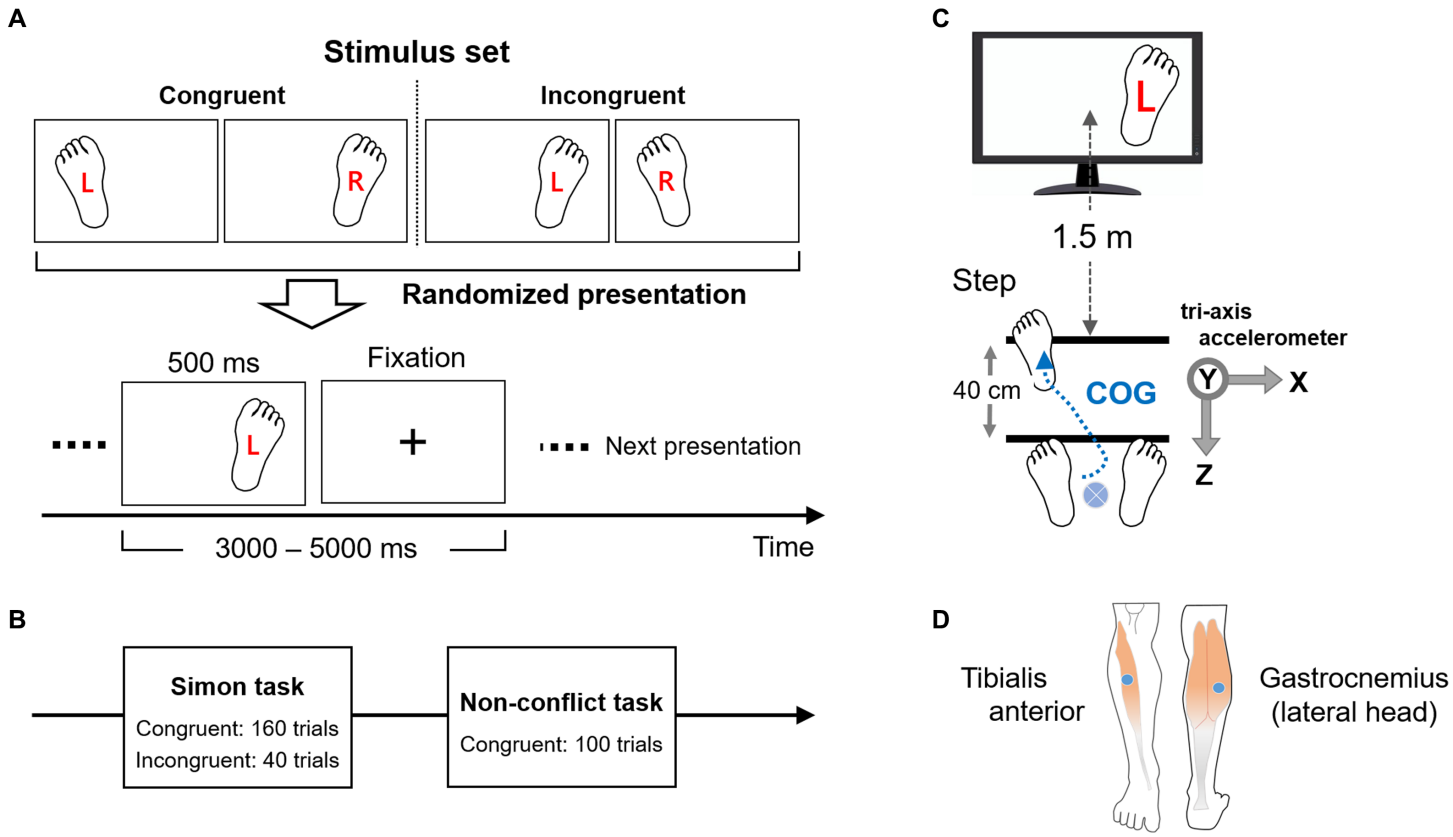
Experimental design and procedure. **(A)** Illustration of the four images used as stimuli in the Simon task (2 congruent and 2 incongruent). The pictures were presented for 500 ms in a random sequence and at a random interval of 3,000–5,000 ms. A fixation cross was displayed during the inter-trial period. **(B)** Outline of the experimental task sessions. In the first session (Session I) was a Simon task consisting of 80% of trials presented congruent stimuli and 20% incongruent stimuli. Two hundred trials were presented in total. The second session (Session II) was a control (non-conflict) task consisting of 100 congruent trials. **(C)** Experimental setup. The Simon stimuli were presented visually using a 17-inch display 1.5 meters from the standing subject. Two lines, 40-cm apart, were drawn on the floor to guide appropriate stepping behaviors, which were monitored by an acceleration sensor. The trial shown is incongruent, with left (L) foot movement required despite presentation of a right footprint. **(D)** EMG sensors were attached to the tibialis anterior and gastrocnemius (lateral head) of both legs (right leg shown) for evaluating EMG activities during tasks and compared to motor and EEG responses. COG: Center of gravity.

The step-motion Simon task was conducted using single left or right footprint images on the right or left sides of a display screen labeled “R” or “L” (Figure 3A). Images were either congruent or incongruent according to the footprint position and label. Subjects were instructed to move the left or right foot 40-cm forward in accordance with the letter (R for the right foot and L for the left foot), but the footprint image could be a left or right foot in accord (congruent) or in conflict (incongruent) with the symbol. The four possible image types were presented in a random sequence, each for 500 ms, at randomly varying inter-trial intervals from 3,000 to 5,000 ms. During inter-trial intervals, a fixation cross was displayed at the center of the screen. Experiments consisted of two sessions (Figure 3B). The first session (Session I) was the Simon task with 200 trials in total, including 160 congruent and 40 incongruent trials. The second session (Session II) was a non-conflict (NC) task including only 100 congruent trials. All images were presented on a 17-in liquid-crystal display placed 1.5 m from the standing participant (Figure 3C). Image presentation was controlled by BioTrace+Software for Nexus32 (MindMedia, Inc). The subjects were allowed to rest between sessions to consider fatigue while performing the task.

### 2.3 Measurements and analyses

#### 2.3.1 Data recordings

Behavioral task performance was evaluated by reaction time (RT) and error responses (i.e., irrelevant responses). RT was evaluated from the horizontal component of ACC data (sampling rate: 256 Hz) using a tri-axial accelerometer sensor attached to the back of the lower trunk (L3 spinous process). The ACC provided two reaction stages, the first corresponding to the APA necessary for step-motion and the second to the initiation of foot-off (McIlroy and Maki, 1993; MacKinnon et al., 2007; Santos et al., 2010). Error responses for each trial condition were evaluated by analyzing each frame (static image) of the acquired video records. An EEG was acquired simultaneously using a head cap with 21 Ag/AgCl electrodes of low contact impedance (<5 kΩ) arranged according to the international 10–20 system. The EEG was recorded during all task sessions using a 24-bit EEG system with 256 Hz sampling (Nexus32, MindMedia Inc.). On-line EEG acquisition was conducted via optical-fiber transmission to suppress electrical noise. The acquired EEG data were re-referenced to mean mastoid signals at sites A1 and A2. EMG was used to evaluate ankle muscle activity during the task. EMG sensors were attached to the tibialis anterior and gastrocnemius (lateral head) of both legs (Figure 3D) according to the conventional anatomical viewpoint of step motion (Zipp, 1982; Rainoldi et al., 2004). EMG signals from the muscles were acquired independently and simultaneously from both legs at 1024 Hz. ACC, EEG, and EMG were measured synchronously on the same system (Nexus32).

#### 2.3.2 Data analysis

Data were analyzed separately for each trial condition. In this study, four trial conditions were defined according to C(C), C(I), I(I), and I(C) as sequential congruency. These means current trial (previous trial), C and I refer to congruent and incongruent stimuli. All time-series data, including acceleration, EEG, and EMG, were anchored by event-trigger markers corresponding to sequential image condition and performance (relevant or irrelevant), such as C(C)-relevant. Hence, there were eight distinct event conditions (Table 1). Time-series data were analyzed separately for the eight event conditions (Table 1). Briefly, we extracted epochs from 500 ms before (−500 ms) to 2000 ms on the basis of stimulus onset at time 0. Such time-series data segmentations yielded eight time-locked event-related (ER) traces corresponding to eight event conditions, including ACC, EEG, and EMG.

**Table 1:** Event conditions integrating trial and performance conditions.

	Trial condition			Performance condition
	Current congruency	Prior congruency		
C(C)	Congruent	Congruent		
I(C)	Incongruent	Congruent	×	Relevant
I(I)	Incongruent	Incongruent		Irrelevant
C(I)	Congruent	Incongruent		

ACC data was processed using a low-pass filter (5-Hz cutoff). ER-ACC traces derived from the acceleration data were then used to calculate two reaction times for each subject: the ACC waveform of the horizontal component during step motion can be expressed as a sine curve. The first peak in this curve represents the APA, and the second represents the foot-off. Prior to step initiation, APAs accelerate the center of mass forward and laterally over the stance leg by moving the center of pressure posteriorly and toward the swing leg. Using this feature, we could evaluate the kinesiological and physiological changes that occur during the transition from a quiet standing to the first step following a stimulus of the experimental tasks (Gazit et al., 2020). The RTs of APA and foot-off were calculated by evaluating the time of peak appearance. Hence, APA and foot-off correspond to step preparation and step initiation, respectively. The grand averages of APA and foot-off step initiation RT for the 17 participants were then calculated for each of the eight event conditions. Error responses were considered when both toe and heal parts of the irrelevant step side rose from the floor; the response is regarded as irrelevant. Error responses for each sequential congruency were first evaluated as error rates (dividing the number of errors by the number of trials per sequence) for individual subjects, and averaged thereafter.

We utilized ERPs to examine the whole word processing framework during the Simon task. The cognitive processing for the Simon stimuli, regarded as general ambiguous stimuli, consists of two dissociable stages, i.e., orthographic and phonological processes: the former could capture congruency information, while the latter could capture contextual meaning, including the task goal. As in previous ERP studies addressing such ambiguity (Joos et al., 2020; Imbir et al., 2022), the former process provides the P200 reflecting congruency perception, while the latter provides the N200 reflecting emotional information, the P300 reflecting attentional processing onset, the N400 reflecting contextual processing, and the late positive potential (LPC) or P600 reflecting the profound meaning, including the task goal. Hence, we focused our attention on these ERP components. We set spatial and temporal regions of interest (ROIs) for identifying ERP components in accordance with previous studies (Joos et al., 2020; Imbir et al., 2022). The vertex Cz electrode was selected (Joos et al., 2020), and the time windows were set as follows: 150–275 ms (P200), 200–350 ms (N200), 300–400 ms (P300), 250–550 ms (N400), and 450–800 ms (LPC or P600). In the current study, we focused our interest on the P200 component as a marker of congruency without any higher cognitive processing. Therefore, EEG time-series data were elicited from the Cz electrode and bandpass-filtered (0.05–45 Hz). Eight ERP waveforms were grand averaged across the 17 participants in accordance with each event condition (Table 1). All time-locked waveforms to the stimulus onset were baseline corrected using averages ranging from −500 to 0 ms.

The EMGs are considered top-down signals from the motor cortex to the peripheral muscles (Hallett et al., 2021). This suggests that the EMG reflects processing in central motor control systems. Hence, we checked event-related EMG traces for evaluating motor control-specific brain activity to support ER-DBA and ERP data. Generally, EMG signals are bipolar, and their average is zero. Hence, we utilized the EMG power signals derived from low EMG signals. Here, we measured two EMG signals from two different muscles per leg, i.e., (EMG_1_) and (EMG_2_), which were transformed to a single EMG power signal by averaging as,


$$EMG_{\mathrm{power}}=\sqrt{\frac{RMS{\left(EMG_{1}\right)}^{2}+RMS{\left(EMG_{2}\right)}^{2}}{2}}$$

(EMG_1_: tibialis anterior, EMG_2_: gastrocnemius, RMS: root mean square)

We evaluated the RMS-EMG amplitude for the left and right legs separately and calculated the total EMG power. As the step motion can be characterized by the muscles, i.e., tibialis anterior and gastrocnemius, the total EMG power reflects higher-order motor control commands such as GO or STOP. In the current study, the 24-bit time-series EMG data were filtered using a third-order Butterworth IIR bandpass filter with a frequency window of 20–500 Hz and transformed to RMS amplitude time-series data before transforming the two RMS-EMG amplitude time-series data into a single EMG power time-series data. We further evaluated eight grand-average EMG traces time-locked to the stimulus onset using the eight event-marker sets used in ERP trace calculations. These analyses were performed separately for the support and step sides during the task. The reliability of the ER-EMG power traces was confirmed by the standard error of the mean (SEM). Furthermore, muscle activity-related characteristics were evaluated during the stepping motion by determining the difference (Δ) in EMG power between the stepping and supporting sides.

#### 2.3.3 Event-related deep-brain activity (ER-DBA) method

The study on the brain regions correlated with relationships between occipital EEG alpha powers and cognitive and emotional activities originates from the past (Larson et al., 1998; Sadato et al., 1998; Lindgren et al., 1999; Goldman et al., 2002; Schreckenberger et al., 2004; Oishi et al., 2007). These studies revealed that the occipital EEG alpha power correlates contain various regions, including the limbic system and upper brain stem. Alpha oscillations may be related to generators of cortical rhythm, such as the occipital cortex (Salek-Haddadi et al., 2003). Recent results established that the faster (>0.1 Hz) and slower (<0.04 Hz) components of the occipital EEG alpha-2 (10–13 Hz) power fluctuations reflect the activities of the dACC and the VTA containing dopaminergic neural systems, respectively (Omata et al., 2013).

This finding has been extended to the development of the ER-DBA method and evaluating the dynamic dACC activity during word-generation (Imai and Katagiri, 2018) and memory consolidation tasks (Araki et al., 2018). These studies revealed that ER-DBA increases reflect improved dACC-DLPFC and dACC-SMA connectivity via the caudal dACC, where the former connection is associated with seeking external information and the latter connection is associated with execution; in contrast, a decrease implies rostral dACC-mPFC connectivity for internal processing, including memory recall and value evaluation. Hence, typical cognitive control processing provides a characteristic ER-DBA waveform accompanied by a dip, whose resolution corresponds to proactive dACC decision-making. The previous two studies also ascribed failure responses during cognitive tasks to a lack of ER-DBA dips reflecting reactive decision-making of the dACC. Hence, dip depth reflects differences in the cognitive cost necessary for promoting such proactive and reactive control.

The ER-DBA method is similar to conventional event-related desynchronization/synchronization (ERD/ERS) techniques (Sochůrková et al., 2006; Wianda and Ross, 2019; Zhozhikashvili et al., 2022). However, only the ER-DBA method can provide dynamical information about fast dACC activity, as supported by EEG-fMRI simultaneous measurements (Omata et al., 2013).

Based on these previous studies, we adopted the ER-DBA method for this study. First, occipital EEG amplitude signals elicited from the O1 and O2 electrodes referring to mastoid electors (A1 and A2) were filtered using a third-order Butterworth digital filter to extract the alpha2-band component from the signals and smoothed by a moving average (time window: 32.25 ms). We defined a DBA index as the mean actual power of these amplitudes. The O1 and O2 mean power data were averaged and further segmented into epochs from −500 to 2000 ms around stimulus onset, according to eight event conditions. Then, ER-DBA waveforms were grand averaged and baseline corrected using averages of the prior epoch ranging from −500 to 0 ms. Each grand averaged ER-DBA waveform time-locked to stimulus onset was statistically evaluated using SEM. Differences between ER-DBA traces (Δ ER-DBA traces) were further examined to evaluate the features of dACC activity specific to the stimulus conditions. Notably, we focused on the differences as NC – C(C) for evaluating the dACC activity specific to the Simon task, I(I) – C(C) to evaluate the one necessary for active inference during the Simon task, and C(I) – C(C) to evaluate the congruency sequence effect.

### 2.4 Statistical analysis

Statistical analyses were performed using Origin Pro 2022 software (OriginLab, Northampton, MA, USA) to examine the statistical significance among the event conditions for each index. The Kruskal–Wallis test was used to analyze the error rate, and the Dunn test was conducted for post-hoc analysis. The RTs of APA and foot-off were analyzed using two-way ANOVA (trial conditions × eight performance conditions). ERPs were analyzed using two-way ANOVA (ERP components × congruent or incongruent trials). When an interaction effect was observed in the two-way ANOVA, the Sidak–Holm test was conducted as a *post hoc* test. ER-DBA depth [depths for C(I), C(C), I(C), and I(I)] was analyzed using one-way ANOVA, and the Sidak–Holm test was used to examine sequential effects. Statistical significance was set at *p* < 0.05. Time-series traces (ACC, EEG, and EMG) were characterized by peak latency and amplitude and are expressed as mean ± standard error.

## 3 Results

### 3.1 Summary of Simon task performance

**Figure 4:**
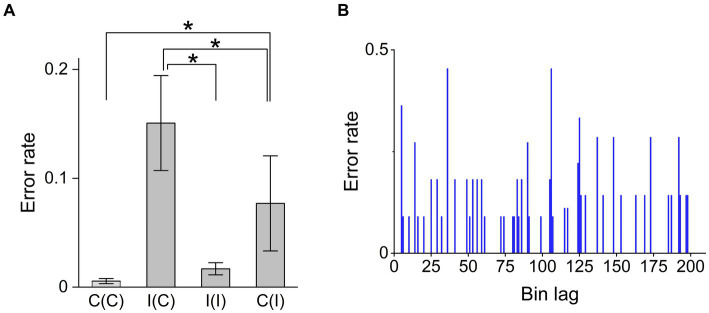
Factors influencing Simon task trial accuracy. **(A)** Error rates for the trial sequence conditions congruent–congruent [C(C)], incongruent–congruent [I(C)], incongruent–incongruent [I(I)], and congruent–incongruent [C(I)]. The Dunn test, as a part of the Kruskal–Wallis ANOVA ( $\chi^{2}$ = 15.5, $p>\chi^{2}=0.0014)\text{,}$ identifies pairs with significant differences in error rate as C(C)-C(I) (*p* = 0.0026, *Z* = −3.5), C(I)-I(C) (*p* = 0.0088, *Z* = −3.2), and I(C)-I(I) (*p* = 0.034, *Z* = 2.8), suggesting that the error rate was significantly higher when switching the congruency condition on consecutive trials compared to non-switching consecutive trials. **(B)** Error rate demonstrated no trend over the course of a session, indicating that error occurrence was idiopathic (not due to fatigue). “*” means statistically significant difference between conditions (*p* < 0.05).

The Dunn test, a *post hoc* test of Kruskal–Wallis test (*χ*^2^ = 15.4, *p* = 0.0014), indicated that the error frequency in the Simon task strongly increased from congruent to incongruent trials (*p* = 0.0026, *Z* = −3.5) and decreased in repeating incongruent trials (*p* = 0.0034, *Z* = 2.7; Figure 4A). Other combinations provided no significant differences. The error frequency did not significantly increase when congruency changed from incongruent to congruent trials (*Z* = −0.418). The results were consistent with the prediction (2) that the error frequency could decrease due to the higher cognitive load.

Here, we found that errors occurred randomly during the session, suggesting no effects of habit or fatigue (Figure 4B). This suggests that the ARC model does not utilize any memory effects associated with the Bayesian surprise term.

**Figure 5:**
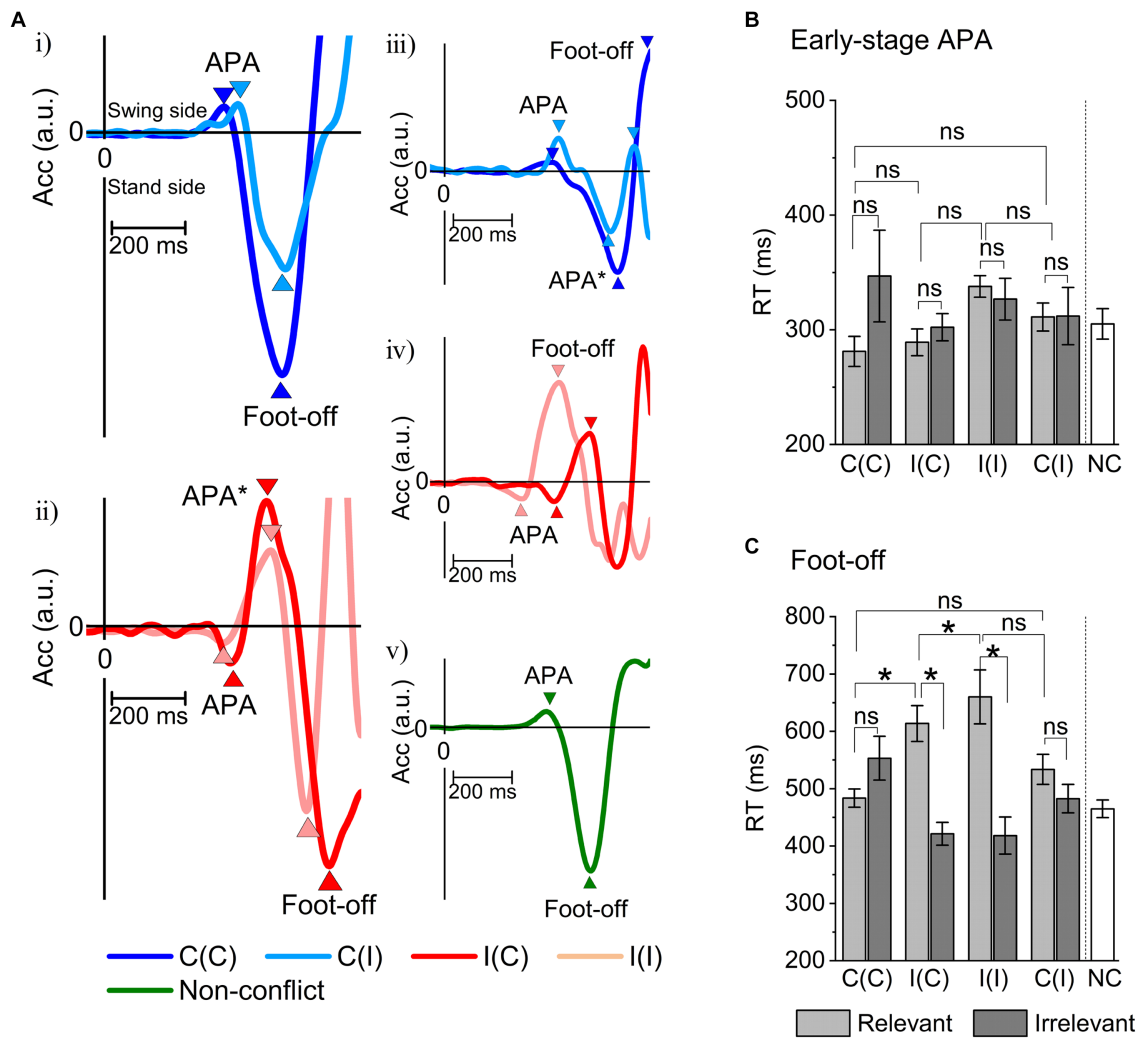
Measurements of peak early-stage anticipatory postural adaptation (APA) and behavioral reaction time (foot-off RT) under all event conditions derived from ER-ACC traces. **(A)** Examples of acceleration traces along the *X*-axis (horizontal to the stepping direction) averaged for each subject: (i) C(C)- and C(I)-relevant; (ii) I(C)- and I(I)-relevant; (iii) C(C)- and C(I)-irrelevant; (iv) I(C)- and I(I)-irrelevant; (v) Non-conflict (NC) task. These traces revealed early-stage APA at ~300 ms during relevant responses, all of which were consistent with location information and independent of congruency. Foot-off times were delayed by around 100 ms under incongruent conditions [I(C) and I(I)] compared to congruent conditions [C(I) and C(C)]. The acceleration traces during relevant responses under incongruent conditions exhibited smooth reversal for goal-directed behavioral correction. The acceleration traces with no correction under incongruent conditions indicate execution while maintaining the irrelevant APA. These ACC trace features were shared by all subjects. In contrast, an example of ER-ACC trace for the NC task (Session II) yielded an ER-ACC trace exhibiting clear APA and foot-off times. **(B)** Summary of grand-averaged peak APA time for the four sequence conditions C(C), I(C), I(I), and C(I) and performance classes (relevant and irrelevant). The two-way ANOVA showed no significant sequential differences (*F* = 1.4, *p* = 0.23) nor a significant difference between relevant/irrelevant difference (*F* = 1.8, *p* = 0.17). Hence, the Sidak–Holm test performed after a two-way ANOVA identified no pairs with a significant difference in reaction time. **(C)** Summary of grand-averaged foot-off times for event conditions. The two-way ANOVA showed a significant sequential difference between relevant and irrelevant responses (*F* = 15.6, *p* = 0.00018) but no significant sequential difference (*F* = 0.18, *p* = 0.89). The Sidak–Holm test after a two-way ANOVA identified six pairs with a significant difference in reaction time: irrelevant I(C)-relevant I(I) (*p* = 0.000000103), irrelevant I(C)-relevant I(C) (*p* = 0.0000098), relevant I(I)-relevant C(C) (*p* = 0.000041), irrelevant C(I)-relevant I(I) (*p* = 0.00027), irrelevant I(I)-relevant I(I) (*p* = 0.00047), and relevant I(C)-relevant C(C) (*p* = 0.0019). *p < 0.05, APA*: corrected APA by reversed.

Next, the early and late RTs (reflecting APA and foot-off delay, respectively) for each performance and stimulus condition (relevant/irrelevant × 4 event conditions) in Session I were evaluated using individuals’ grand-averaged ER-ACC traces (Figure 5A). Session II was analyzed only with congruent stimuli and relevant trials (no errors occurred). The APA and foot-off times were clearly distinguishable in all traces (event conditions) as well as the NC condition, but the time to peak APA was generally longer and the foot-off delay was usually shorter in incongruent trials (Figures 5B,C).

We conducted multiple comparisons using the Sidak–Holm test after the two-way ANOVA to identify pairs under eight performance and stimulus conditions providing significant differences in RTs of the early-stage APAs, but no pairs were found (the C(C)-I(I) pair provided the minimal probability of 0.12). These results are consistent with our third theoretical prediction (Figure 5B).

In contrast, similar ANOVA analyses on the RTs of the foot-off identified pairs with significant differences: irrelevant I(C)-relevant I(I) (*p* = 0.000000103), irrelevant I(C)-relevant I(C) (*p* = 0.0000098), relevant I(I)-relevant C(C) (*p* = 0.000041), irrelevant C(I)-relevant I(I) (*p* = 0.00027), irrelevant I(I)-relevant I(I) (*p* = 0.00047), and relevant I(C)-relevant C(C) (*p* = 0.0019; Figure 5C). Among them, the relevant/irrelevant I(C)-I(C) pair was notably attributed to the absence of active inference. This was supported by the lack of significant differences in the irrelevant I(C)-relevant C(C) pair, suggesting that the irrelevant I(C) response was as fast as that for the relevant C(C) pair. In contrast, the irrelevant C(I)-relevant I(I) pair significantly differed, suggesting that erroneous responses for the congruent trials could be promoted by response conflict. This result was consistent with our second prediction.

### 3.2 Effects of Simon task conditions and performance on DBA and ERPs

**Figure 6:**
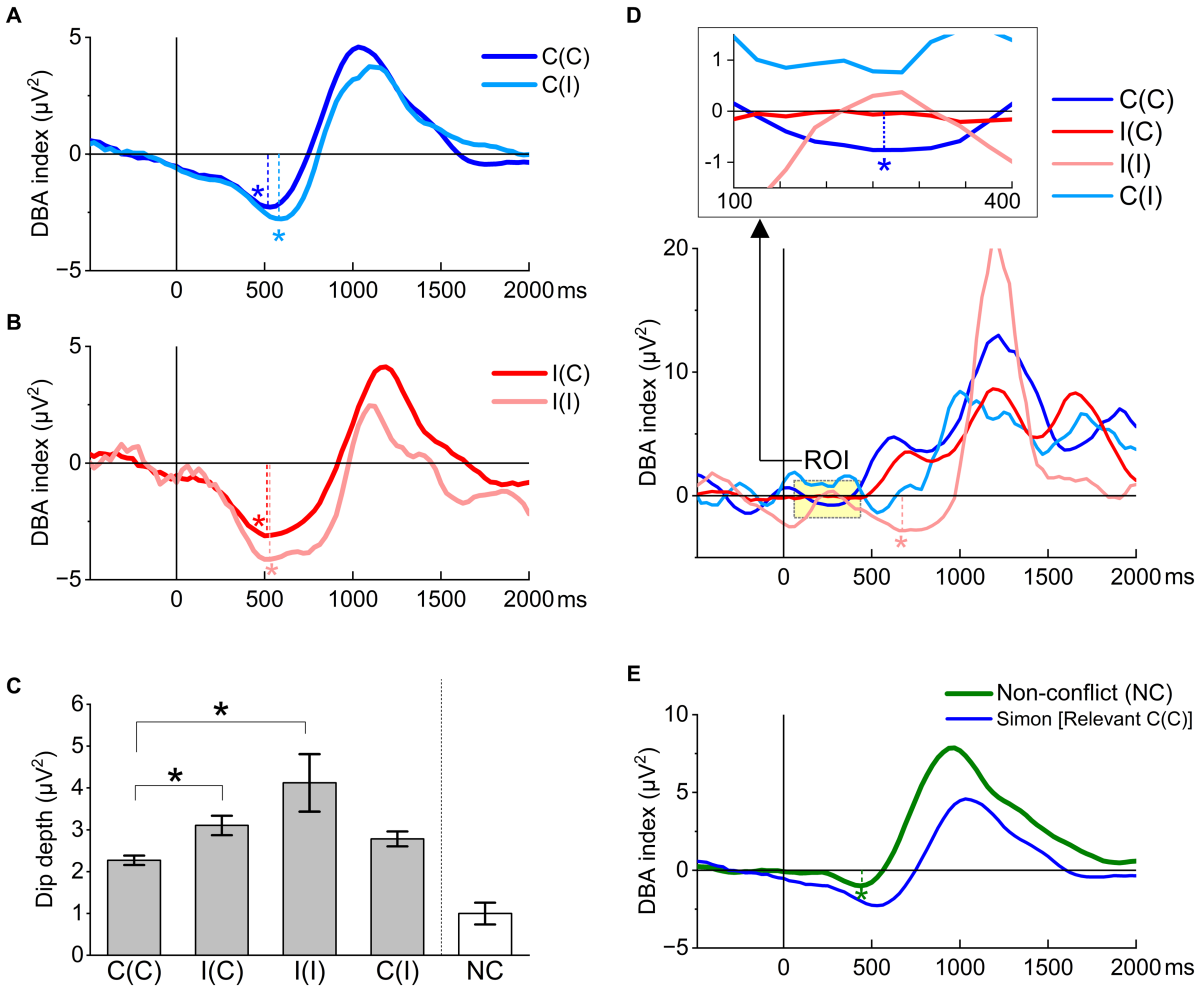
Task performance effects on ER-DBA traces locked to stimulus onset. **(A)** ER-DBA traces recorded during relevant C(C) and C(I) trials. Both traces exhibited a large negative waveform (dip, asterisk) beginning later than 300 ms post-stimulus onset. This dip was slightly attenuated in the successive congruent condition C(C) compared to the switching condition C(I). **(B)** ER-DBA traces recorded during relevant incongruent trials I(C) and I(I). The dip depth was much larger for the successive condition I(I) than the switching condition I(C), while both traces reach a minimum at similar latencies of around 500 ms post-stimulus onset. Further, dip depths were much larger than those in the C(C) and C(I) conditions. **(C)** Summary of sequence effects on the DBA dip depth. Dip depths greatly differed when the congruency changed. The Sidak–Holm test after the one-way ANOVA for the four congruency conditions limited to relevant responses identified two pairs with significant differences in dip depth: I(C)-C(C) (*p* = 2.4E-4) and I(I)-C(C) (*p* = 0.0077). *p < 0.05 **(D)** ER-DBA traces recorded during irrelevant responses. The trace exhibited an early dip around 300 ms only for the successive congruent condition C(C) (within the region of interest [ROI] shown in the inset above), while only the I(I) condition exhibited a late-stage dip around 600 ms. **(E)** The NC task exhibited a much smaller DBA dip at around 450 ms, corresponding to the foot-off time.

ER-DBA originating from electrodes O1 and O2 was greatly influenced by both Simon task trial sequence (congruency) and response. All grand-averaged ER-DBA traces for relevant responses decreased from the time of stimulus onset and exhibited a dip, reaching a nadir around the grand-averaged foot-off time (Figures 6A,B). This result testified to the prediction (5) that the dACC could be crucial for cognitive processing. Moreover, dip depth was significantly larger (lower) in conditions including an incongruent trial compared to the C(C) condition (Figure 6C).

Based on previous DBA recordings during cognitive tasks, including word generation tasks (Araki et al., 2018) and paired word memorizing and retrieval (Imai and Katagiri, 2018), we believe that the DBA index represents the cognitive cost of executing tasks.

The congruency blocks were provided as C(C)-I(C)-I(I)-C(I) in the Simon task. The dip depths differed when the congruency changed. The Sidak–Holm test after the one-way ANOVA for the four congruency conditions limited to relevant responses identified two pairs with a significant difference in dip depth as I(C)-C(C) (*p* = 0.00024) and I(I)-C(C) (*p* = 0.0077). Such a dip-depth difference between congruent and incongruent trials suggested that the cognitive cost was flexibly mediated trial-by-trial by the dACC, resulting in cost savings.

In contrast, grand-averaged ER-DBA traces recorded during irrelevant responses exhibited no such dip around the foot-off time except for the C(C) condition, which provided a dip (Figure 6D). The absence of ER-DBA dips suggests that dACC deterioration may induce erroneous responses. In contrast, excessive activity of the dACC may generate response conflict, thus dismissing the relevant APA. These findings supported our second prediction.

In the NC task, the grand-averaged ER-DBA trace exhibited a small, significant dip (Figure 6E). This suggests that the dACC may reactively select goal-relevant responses. This situation implies that the internal model is completely consistent with the external response; thus, the Bayesian surprise as an inference error in the NC task is always negligibly small.

**Figure 7:**
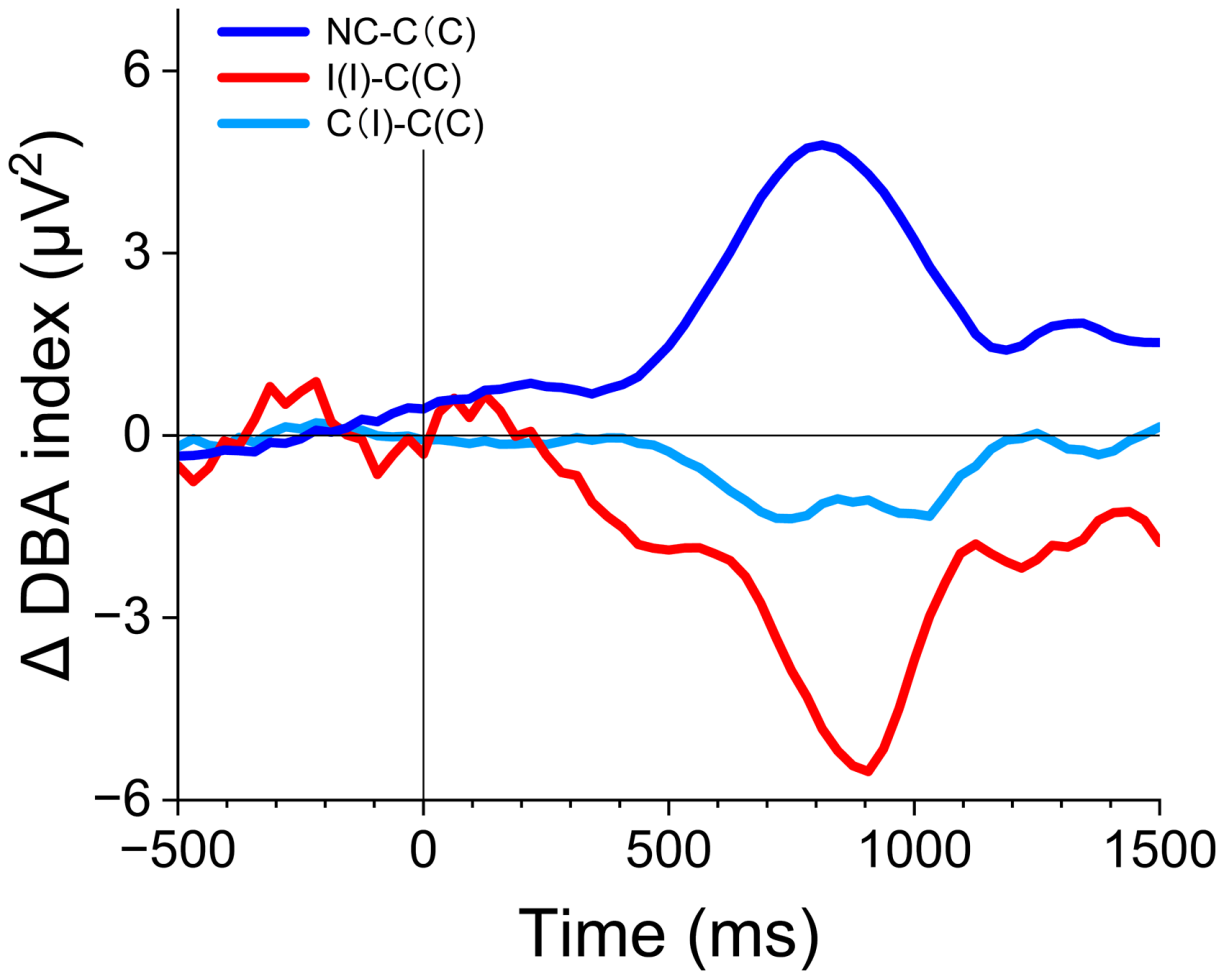
Δ ER-DBA among task and stimulus conditions.

All differences between ΔER-DBA traces except for I(I) –C(C) (Figure 7) showed little deviation from baseline until about 300 ms after stimulus onset (corresponding to P300). The I(I) –C(C) trace started to decrease at around 150–200 ms, suggesting a higher cognitive cost for engaging with incongruent stimuli. However, all Δ ER-DBA traces exhibited substantial trace changes after this critical time point (300 ms).

The large difference between C(C) and NC traces suggests that inhibitory motor control is dominant after this critical time, while the difference between congruent and incongruent trials [C(C) vs. I(I)] suggests higher cognitive load in incongruent trials.

Further, the difference between C(C) and C(I), representing the congruency sequence effect, suggests reduced cognitive load in the successive C(C) condition. This was consistent with the first prediction.

**Figure 8:**
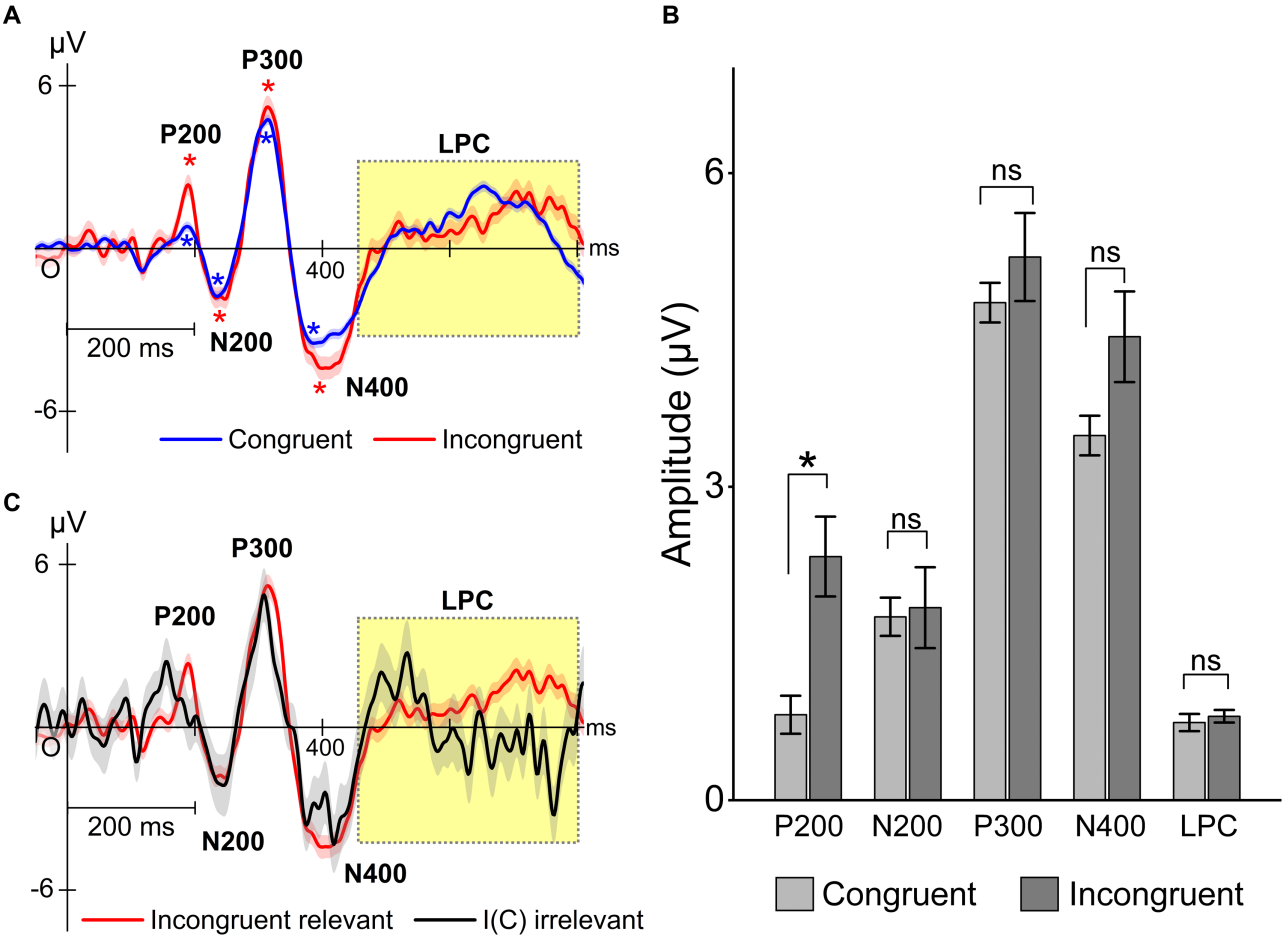
Task performance effects on typical ERPs P200, P300, N400, and LPC (with P600). The ERP traces locked to the stimulus onset were evaluated for congruent [C(C) and C(I)] and incongruent [I(C) and I(I)] conditions. **(A)** ERP traces for congruent and incongruent trials. The asterisk in the figure indicates that the amplitude of the ERP components is significantly larger than that of the baseline (one-sample *t*-test). “*” means significantly different from baseline (*p* < 0.05). **(B)** Statistical evaluation of these differences in relevant responses. Two-way ANOVA showed significant interaction effect (*F* = 5.2, *p* = 0.00035). There were significant differences in the P200 between congruent and incongruent trials using the Sidak–Holm test. “*” means significantly different between congruent and incongruent (*p* < 0.05). **(C)** The ERP traces also differed significantly between relevant and irrelevant responses independently of congruency.

Simon task performance and congruency conditions also influenced the ERPs originating from electrode Cz. Figure 8A shows congruency-specific ERP traces obtained during relevant responses. Traces exhibited typical ERP components P200, P300, and N400, as well as LPC waveforms, including P600. The P200 demonstrated significant differences between congruent and incongruent trials using a post-hoc test (*p* = 0.00012, power = 0.94), and other ERP components were not significantly different (Figure 8B). This result suggests that P200 might reflect congruency, meaning that the fusiform gyrus, as a P200 source, could detect Simon stimuli congruency. This result explanation is consistent with the fourth prediction associated with automatic congruency detection. ERP waveforms were also identified for both relevant and irrelevant responses (Figure 8C). They clearly indicated that the P200 appeared earlier in the relevant condition than the irrelevant condition. The LPC (late positive potential) exhibited an earlier component at around 500 ms in the irrelevant condition and a significant late component at around 700 ms in the relevant condition (*p* = 0.0002). These results suggest that the LPC may reflect successful trial execution.

### 3.3 Associations of Simon task condition and performance with motor activity

**Figure 9:**
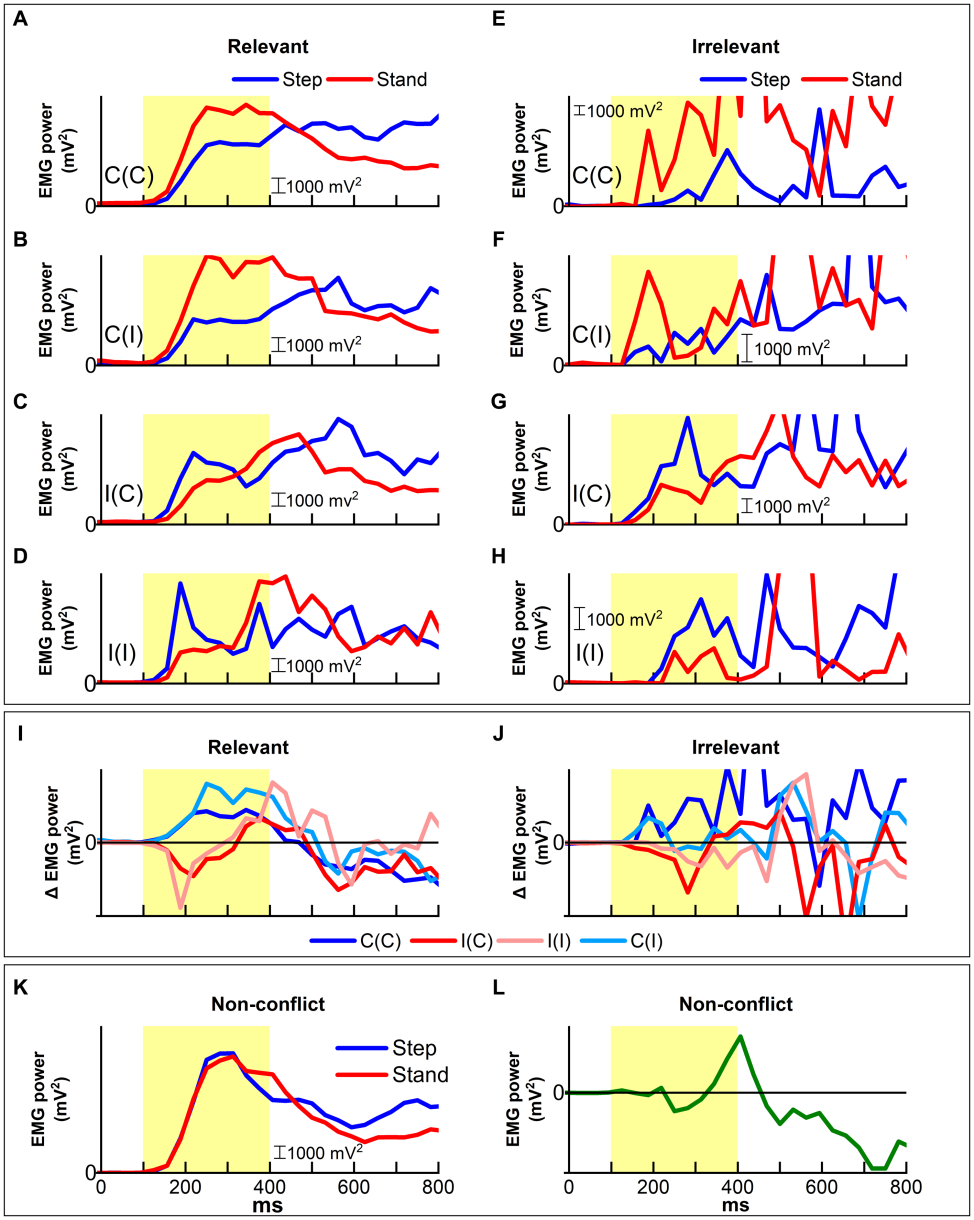
Patterns of muscle activity for leg movement (step) and contralateral support (stand) as measured by ER-EMG traces are influenced by task congruency and performance. **(A–D)** All congruent trial response **(A,B)** and incongruent trial response **(C,D)** showed distinct and lateralized EMG patterns in the early-stage up to 300 ms post-stimulus onset. In all cases, lateralization reflected location information in the Simon trial. **(E–H)** In contrast, ER-EMG patterns for irrelevant responses were rather complex. **(E)** In congruent trials, responses showed that a maintained goal-relevant pattern up to around 600 ms followed by a sudden inversion leading to irrelevant responses. The irrelevant responses for the other conditions **(F–H)** exhibited similar patterns compared to relevant responses. **(I)** ER-EMG trace difference between step and support (stand) sides represented the APA for stepping. The irrelevant APAs were reversed at around 300 ms for response correction. **(J)** In contrast, the irrelevant APAs were not corrected. **(K)** The ER-EMG trace recorded during NC condition showed to lateralization or reversal in the early-stage. **(L)** The difference between step and support sides became substantial only after ~300 ms, implying that automatic processing was not recruited in the effortless NC task.

All ER-EMG traces recorded during the Simon task (Figures 9A–H) exhibited a rise in the range of 100–150 ms post-stimulus onset and clear lateralization of EMG power to the swing side compared to the support side according to location information. The critical time of 150 ms corresponded to the ERP P200 component. The emergence of EMG power indicated that motor control processing started very early, at least up to 150 ms.

Among the relevant responses (Figures 9A–D), EMG traces time-locked to the stimulus onset for the congruent condition peaked at 200–250 ms post-stimulus onset. Notably, EMG power on the step side provided steep negative inclinations for the incongruent condition at around 200 ms post-stimulus onset, which corresponded to the ERP P200 component. After the peak, the EMG trace on the swing side steeply decreased, whereas that on the support side increased in the incongruent trials. This lateralization of EMG power toward the support side denoted that motor control processing started to guide goal-irrelevant APAs to goal-relevant responses. In contrast, such EMG power lateralization was not exchanged in congruent trials.

The EMG power increased in both congruent and incongruent trials after 300 ms post-stimulus onset. This critical time corresponded to the ERP P300 component, whose source is the ACC. The lateralization of EMG power reversed at around 400–500 ms, corresponding to the ERP N400 component in incongruent trials, whereas it maintained the same pattern emerging at the early stage in congruent trials. Interestingly, the EMG patterns in the incongruent condition, which were promptly corrected from 200 to 350 ms, manifested the same pattern as in the congruent condition.

In contrast, irrelevant responses (Figures 9E–H) exhibited patterns similar to relevant responses at earlier stages. However, the EMG traces were not corrected until around 450 ms post-stimulus onset in the incongruent condition, while traces in the congruent condition exhibited earlier corrected patterns.

Figure 9I shows the Δ EMG trace difference for relevant responses during the Simon task to evaluate the magnitude of EMG lateralization. The difference increased with time post-stimulus onset but manifested distinct features according to congruency. For the congruent condition, the difference peaked at around 250 ms and then gradually decreased, while in the incongruent condition, the difference exhibited a sharp reversal at 200 ms, and reached almost the same level as that in the congruent condition at around 400 ms post-stimulus onset. Such reversal for relevant responses was not observed for irrelevant responses in the incongruent condition (Figure 9J).

The ER-EMG traces for the NC task (Figures 9K,L) showed no difference between swing and support sides in the early-stage up to 300 ms, which corresponded to the peak times of the ER-EMG traces (Figure 9K). After the peak, traces exhibited the same changes as those for the relevant responses in the congruent condition of the Simon task. However, the peak levels were much higher than those of the Simon task. The difference in ER-EMG between step and support sides during the NC task exhibited a small reversal around 250 ms followed by a rapid increase at around 300 ms.

### 3.4 Evaluation of cognitive processing in the dACC

We evaluated Δ ER-DBA traces between task conditions for extracting process-specific cognitive costs (Figure 7). The I(I) – C(C) difference corresponded to the cognitive costs associated with Simon-task-specific processing. The difference started at around 200 ms post-stimulus onset, suggesting that cognitive processing for engaging in incongruent trials was initiated by congruency detection in the dACC. In contrast, the C(I) – C(C) difference corresponded to the cognitive cost reduction by repetition of congruent trials. The difference started at around 500 ms post-stimulus onset. This suggests lower incongruence-specific cognitive cost in C(C) trials. The foot-off times, as reaction times, were also around 500 ms for the C(C) trials. Hence, the incongruence-specific cognitive cost should be associated with decision-making in the dACC. Cost-saving implies that cognitive control may be more reactive in the C(C) trials. Overall, these considerations are consistent with the ARC model referring to the FEP. We further examined the Δ ER-DBA of NC – C(C) and found that the cognitive cost was much higher for the Simon task than for the NC task. This cognitive cost increase in the Simon task may be attributed to the active inference for engaging in the Simon task.

## 4 Discussion

**Figure 10:**
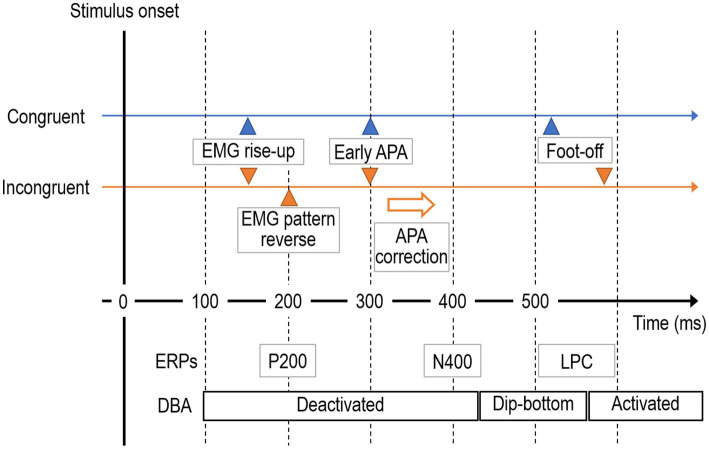
Primal behavioral and neurophysiological events during the step-motion Simon task: summary of results.

We proposed and demonstrated an action-based cognitive control (ARC) model to show evidence that response-conflict tasks can be executed beyond the trade-off between cost saving and accuracy by behavioral adaptation via active inference. Figure 10 summarizes the results, which are consistent with the predictions set in the Introduction section. To confirm the feasibility of the ARC model, we examined whether alternative theories could explain the results.

### 4.1 Response adaptation by ARC versus top-down inhibition of irrelevant responses through a hyper-direct pathway

Alternative theories typically include the conflict monitoring theory (Schmidt, 2019), wherein the ACC monitors conflict and triggers the PFC to suppress irrelevant responses in Simon tasks (Kerns, 2006). This hypothesis claims that the PFC replaces the prior irrelevant response with the relevant response, while the subthalamic nucleus (STN) promotes global (nonselective) motor inhibition, leading to response delay (Wessel et al., 2019). Hence, the hypothesis fits the conventional dual root model (Luo and Proctor, 2019). The hypothesis also claims that the conflict adaptation effect in Simon tasks, i.e., the Gratton effect (Gratton et al., 1992), is part of such global inhibition by the STN (Hommel et al., 2004). However, our results disagree on several points. First, prior irrelevant APA responses in the incongruent trials were reused for generating relevant responses. Furthermore, such behavioral adaptation started at around 200 ms after stimulus onset, which means that the behavioral adaptation observed in our results was not affected by global motor inhibition. Second, although we identified such STN-promoted global inhibition, it was selective. EMG was asymmetrically generated on the right and left sides. This difference was attributed to the EMG inhibition on the step side. This means that spatial information is perceived, at least at this early stage. This difference was attributed to EMG inhibition on the step side. This means that the spatial information is at least perceived at this early stage. We ascribed such selective inhibition to some metacognitive risk-hedge manners. The impulsive reaction based solely on the information brought by the dorsal visual pathway is risky. Hence, the Simon stimuli-triggered fast reaction, notably on the spatial-information-relevant side, and is proactively inhibited by the rostral dACC. Such selective inhibition was observed only in the Simon task but not in a non-conflict task consisting of relevant trials only.

### 4.2 Response adaptation by ARC versus feature integration without executive control

The feature integration theory (FIT) (Mayr et al., 2003; Hommel et al., 2004) has been proposed for accounting sequential effects in Simon tasks by automatic processing without top-down executive control. We thus examined whether the FIT could alternatively account for the results of this study. The theory is based on a bottom-up framework using SR mapping. During Simon tasks, the SR maps must be memorized and recalled trial-by-trial. Such FIT processing framework is certainly advantageous for cognitive cost reduction for conflict resolution. However, FIT feasibility is still argued (Schmidt et al., 2015) to many options included the maps. To apply such FIT to account for the cognitive processing in Simon tasks, the option selection from the SR maps should appear in a late processing stage after semantic processing for the Simon stimuli. Thereby the FIT could not account for our results of early-stage correction of irrelevant APAs in incongruent trials at approximately 200-ms post-stimulus onset. Furthermore, neural evidence in this study characterized by ER-DBA dips indicates that any executive control association via the dACC disagrees with the FIT characterized by executive control absence. Hence, the flexible dACC activity predicted by the ARC model for prompt response adaptation in the Simon task may explain conventional congruency sequence effects better than the FIT.

### 4.3 Absence of trade-off between cost-saving and accuracy

An important concept of this discussion is that the ARC model could account for the cost-saving effect. Recent studies have reported that the caudal dACC interacts with dorsolateral prefrontal cortex to control impulsive decision-making while the rostral dACC interacts with the OFC to control strategic decision-making (Venkatraman et al., 2009; Venkatraman and Huettel, 2012; Konovalov et al., 2021). It has also been demonstrated that strategic decision-making promoted by the rdACC provides accuracy at the cost of additional cognitive load, while impulsive decision-making promoted by the cdACC provides rapidity at the cost of increased error rate (Kim et al., 2011; Leota et al., 2021). Such duplicate decision-making mechanisms can be either compensatory or conflicting under time pressure, so each strategy has distinct merits and drawbacks (Ebitz and Hayden, 2016). This indicates that there is a trade-off between cognitive cost-saving and accuracy.

In contrast, a major portion of the total cognitive cost of the Simon task may be allocated to information processing, which is inversely correlated with free energy (Trujillo, 2019). Accordingly, the cognitive cost was necessary for the active inference to reduce the entropy associated with the Shannon surprise. Our results indicate that the ER-DBA dip depth changed in accordance with stimulus sequence, with the greatest depth for the successive incongruent condition and the shallowest for the successive congruent condition. Such trial-by-trial adaptation suggests that cognitive costs may be actively regulated in accordance with circumstances.

Such flexibility enables not only cost-saving but also cost-saving and accuracy beyond their trade-off relationship as described by previous studies (Marshall et al., 2006; Klein-Flügge et al., 2016; Shenhav et al., 2016). Of course, the ARC model does not completely prevent the risk of cognitive errors. For successive congruent trials, such cost-saving degrades the activity of the rostral dACC due to the disuse of active inference, which counterbalances the promotion of impulsive decisions mediated by the caudal dACC. This increases the error risk when the trial changes to incongruence as I(C). However, such a risk will be avoided if the dACC maintains high flexibility while being sufficiently supported by the ventral tegmental area involving abundant monoaminergic neurons.

### 4.4 Limitation

It is unclear whether this ARC model can be extended to other task paradigms. In theory, a simple APA reversal mechanism utilizing the spinal central pattern generator (McCrea and Rybak, 2007; McCrea and Rybak, 2008; Danner et al., 2015; Minassian et al., 2017; Merlet et al., 2021; Ryu and Kuo, 2021) and midbrain locomotion area (Shefchyk and Jordan, 1985; Noga et al., 2003, 2017) could regulate step-motion conflict based on mature walking mechanisms. If so, our findings were task specific. Moreover, while our study provides data supporting the ARC model, we did not directly compare or test this model against other existing models, which is a limitation that should be considered when interpreting our results. The current study also did not examine patients with neuropathological disorders such as literal spatial neglect. It is debated whether allocentric spatial reference frameworks exist independent of egocentric references (Filimon, 2015; Demeyere and Gillebert, 2019). The experimental paradigm described here could be adapted to examine how these patients resolve Simon-like SRCs, which may in turn provide clues to the underlying path mechanisms. For instance, these patients could be examined in Simon-like tasks using virtual-reality (Vourvopoulos et al., 2016; Laver et al., 2017; Riva et al., 2019; Hoffman et al., 2020; Sip et al., 2023) or brain-machine interface technologies (Juliano et al., 2020; Leeb and Pérez-Marcos, 2020).

## 5 Conclusion

In this study, we proposed a novel ARC model where Simon stimulus information is reduced from 2-to 1-bit by categorizing Simon stimuli into simple congruent/incongruent groups (Prezenski et al., 2017; Bejjani and Egner, 2019). We theorized the ARC model based on the free energy principle to show the validity of the ARC model by making theoretical and experimental predictions. One of our main predictions is that the simple behavioral adaptation rule of guiding automatic APAs to goal-relevant responses could be promoted by active inference. Furthermore, we predicted that congruency repetition blocks contribute to cost-saving. We conducted a step-motion Simon task with simultaneous EEG, EMG, and kinetic measurements to investigate neural mechanisms, hypothesizing that flexible dACC activity would be crucial for realizing the ARC model. Our results were consistent with those theoretically predicted, suggesting ARC model feasibility. Taken together, we could conclude that the ARC model provides rationale for modifying complex stimulus–response mapping processes to more sophisticated rule-based processing characterized by simplicity and cost-saving. This model could potentially aid in cognitive training and rehabilitation program development. In this study, the FEP was formulated, and model validation was conducted. Behavioral modifications due to active inference were observed during step movement during the Simon task. Since this finding may be task specific, it would be possible to discuss generalization, including model extension, by verifying whether active inference is also valid for different types of movements, such as upper limb movements and finger tapping.

## Data availability statement

The raw data supporting the conclusions of this article will be made available by the authors, without undue reservation.

## Ethics statement

The studies involving humans were approved by Ethics Committee of Kobe University Graduate School of Health Sciences. The studies were conducted in accordance with the local legislation and institutional requirements. The participants provided their written informed consent to participate in this study.

## Author contributions

YO, YK, EI, and HK contributed to the conception and design of the study and critically reviewed the manuscript. YO performed the manuscript and data collection and analyses. YO and YK wrote the first draft of the manuscript. All authors contributed to manuscript revision and approved the submitted version.

## Figures and Tables

**Table:** 

APA	Anticipatory postural adaptation
COG	Center of gravity
dACC	dorsal anterior cingulate cortex
DBA	Deep-brain activity
DLPFC	dorsolateral prefrontal cortex
EEG	Electroencephalography
EMG	Electromyogram
ER-ACC	Event-related acceleration
ER-DBA	Event-related deep-brain activity
ER-EMG	Event-related electromyography
ERP	Event-related potential
FG	Fusiform gyrus
LPC	Late positive complex
mPFC	Medial prefrontal cortex
OFC	Orbit frontal cortex
PMd	dorsal premotor cortex
RT	Reaction time
SMA	Supplemental motor area
SRC	Stimulus–response conflict
NC	Non-conflict
